# Systemic CD4 Immunity and PD-L1/PD-1 Blockade Immunotherapy

**DOI:** 10.3390/ijms232113241

**Published:** 2022-10-31

**Authors:** David Escors, Ana Bocanegra, Luisa Chocarro, Ester Blanco, Sergio Piñeiro-Hermida, Maider Garnica, Leticia Fernandez-Rubio, Ruth Vera, Hugo Arasanz, Grazyna Kochan

**Affiliations:** 1 Oncoimmunology Group, Navarrabiomed, Fundación Miguel Servet-Hospital Universitario de Navarra-UPNA-IdISNA, Irunlarrea 3, 31008 Pamplona, Spain; 2 Department of Medical Oncology, Hospital Universitario de Navarra, IdISNA, Irunlarrea 3, 31008 Pamplona, Spain

**Keywords:** T lymphocytes, immune checkpoint, biomarker

## Abstract

PD-L1/PD-1 blockade immunotherapy has changed the therapeutic approaches for the treatment of many cancers. Nevertheless, the mechanisms underlying its efficacy or treatment failure are still unclear. Proficient systemic immunity seems to be a prerequisite for efficacy, as recently shown in patients and in mouse models. It is widely accepted that expansion of anti-tumor CD8 T cell populations is principally responsible for anti-tumor responses. In contrast, the role of CD4 T cells has been less studied. Here we review and discuss the evidence supporting the contribution of CD4 T cells to anti-tumor immunity, especially recent advances linking CD4 T cell subsets to efficacious PD-L1/PD-1 blockade immunotherapy. We also discuss the role of CD4 T cell memory subsets present in peripheral blood before the start of immunotherapies, and their utility as predictors of response.

## 1. Introduction

There is clearly a “before” and an “after” in cancer immunotherapy. In 2012, the results from clinical trials evaluating the PD-L1/PD-1 blockade were published, uncovering unprecedented efficacies in a wide range of tumors [1,2]. Immunotherapies had been extensively developed for several decades before this time [3,4,5,6,7,8], but never with the efficacies as reported for the PD-L1/PD-1 blockade. This change in paradigm had already started with the development of ipilimumab, a CTLA-4 blocking antibody [9,10,11,12]. Since then, immunotherapies have changed treatment strategies in oncology.

**Figure 1:**
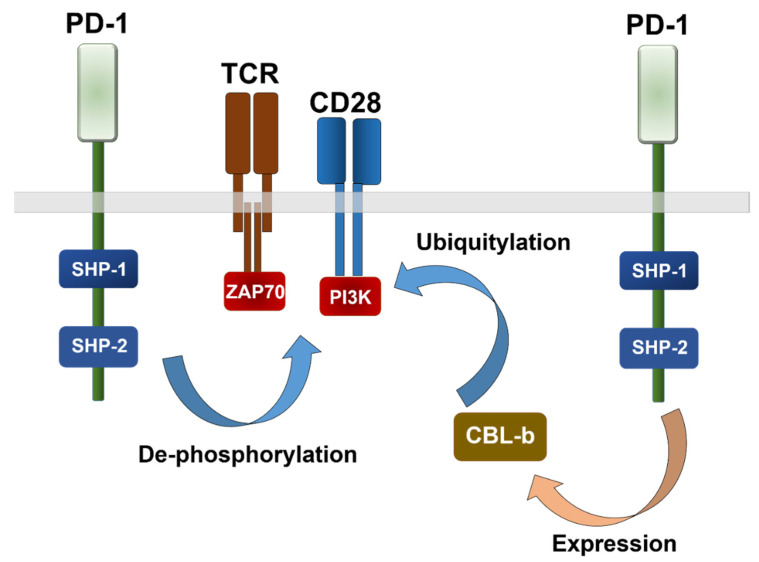
The two main inhibitory pathways of PD-1. A schematic representation of the two main inhibitory pathways regulated by PD-1 in T cells leading to T cell inactivation. On the left, PD-1 after engagement with PD-L1 recruits SHP phosphatases which de-phosphorylate ZAP70, PI3K and additional TCR-dependent signaling kinases. On the right, PD-1 up-regulates the transcriptional expression of the E3 ubiquitin ligase CBL-b, which ubiquitylates components of the TCR signalosome, leading to its internalization and degradation.

PD-L1/PD-1 interactions regulate many immune-related processes, especially T cell responses. In physiological conditions, PD-L1/PD-1 interactions prevent autoreactive damage towards self-tissues [13,14,15], and participate in physiological antigen presentation by regulating the degree of T cell activation [7,14,16]. However, tumors can up-regulate PD-L1 expression to evade T cell responses [17,18]. This up-regulation contributes to the establishment of a systemic state of immunosuppression [19]. Many tumor types respond to anti-tumor T cells by up-regulating the surface expression of PD-L1, which binds to its receptor PD-1 on the surface of tumor-infiltrating T cells (TILs). PD-L1/PD-1 binding then delivers inhibitory signals leading to T cell inactivation through several mechanisms (Figure 1) [17,18,20,21,22,23,24,25].

Most PD-L1/PD-1-targeting drugs are recombinant antibodies that block PD-L1/PD-1 interactions and eliminate T cell inhibitory signals. The first PD-1 blocking agent (pembrolizumab) was approved in 2014 for metastatic melanoma. Since then, several PD-L1/PD-1 blocking antibodies have been approved by the Food and Drug Administration (FDA) for many cancers, which include the PD-1 inhibitors nivolumab, pembrolizumab, cemiplimab and dostarlimab, and the PD-L1 inhibitors atezolizumab, durvalumab and avelumab (Table 1).

Overall, their clinical efficacies are impressive, with durable responses and prolonged survival. However, only a limited number of patients show clinical benefit, while the rest are either refractory or acquire resistance [17,25,26,27,28,29]. Moreover, PD-L1/PD-1 blockade therapies have also been associated with hyperprogressive disease in a proportion of patients leading to death [29,30,31,32,33,34,35,36,37,38,39,40,41]. For these reasons, there is a strong need to identify biomarkers of response that help to discriminate patients according to clinical benefit. As such, the identification of biomarkers for hyperprogressive disease can be equally important.

Although the main mechanisms by which T cell functions are restored through the PD-L1/PD-1 blockade are fairly well-known, immunotherapy failure remains poorly understood. This understanding will be relevant for biomarker discovery and for the development of novel approaches to enhance immunotherapies [42,43,44,45].

There is no doubt that PD-L1/PD-1 blockade antibodies re-activate T cell functions in TILs at the tumor microenvironment [46]. However, recent evidence highlights functional systemic immunity for PD-L1/PD-1 blockade efficacy [29,45,47,48,49,50]. Systemic administration of PD-L1/PD-1 blockers causes broad changes in CD8 T cells that correlate with clinical responses. However, the contribution of CD4 T cells is frequently overlooked even though they are essential regulators of CD8 responses. In this review we discuss systemic CD4 immunity in the context of the efficacy of PD-L1/PD-1 blockade immunotherapy and, more specifically, the status of CD4 immunity before the start of immunotherapies, its influence in efficacy and its relationship with other systemic immune cell types.

## 2. CD4 T Cells and Anti-Tumor Immunity

Anti-cancer immune responses start by the capture and processing of tumor-associated antigens (TAA) by antigen-presenting cells such as dendritic cells (DCs) [5]. TAAs can be varied in nature, and they range from, for example, viral proteins, overexpressed proteins, embryonic antigens and neoantigens. Hence, the nature of such TAAs can affect therapeutic activities and toxicities, as extensively revised in [5]. These TAAs are released by cancer cells dying either by immunogenic cell death or following attack by natural killer cells (NK) through the establishment of an initial inflammatory response. TAA-loaded DCs migrate to secondary lymphoid organs such as lymph nodes, where they prime both CD4 and CD8 T cells specific for these TAAs. As CD8 T cells possess strong cytotoxic activities, this T cell subset has been classically considered as the main effector in anti-tumor immunity through direct tumor killing [51,52,53,54,55]. Following antigen presentation, CD8 T cells expand exponentially in peripheral blood and differentiate into cytotoxic T cells (CTLs). These primed T cells infiltrate tumors where they recognise cancer cells bearing TAAs and exert their cytotoxic activities [7,16,53,56,57]. It is believed that this process of TAA recognition and initial immunological attack over tumors takes place right at the beginning. Thus, most cancers that progress end up evading this initial immunological attack by reducing their immunogenicity through immunological editing. This escape mechanism leads to the selection of poorly immunogenic cancer cell variants which down-modulate MHC molecules, or express immunosuppressive molecules [58,59,60,61,62,63,64].

The CD4 T cell contribution to anti-cancer immunity has been much less studied. Even so, the evidence supporting their anti-tumor capacities is compelling, principally by regulating innate and adaptive immunity [65,66]. Indeed, their importance is highlighted by the study of immunoedited cancer cells, in which mutations in MHC-II-restricted neoantigens are more potently selected during tumorigenesis [67,68,69]. This reflects the significant contribution of CD4 T cells in immunosurveillance.

**Figure 2:**
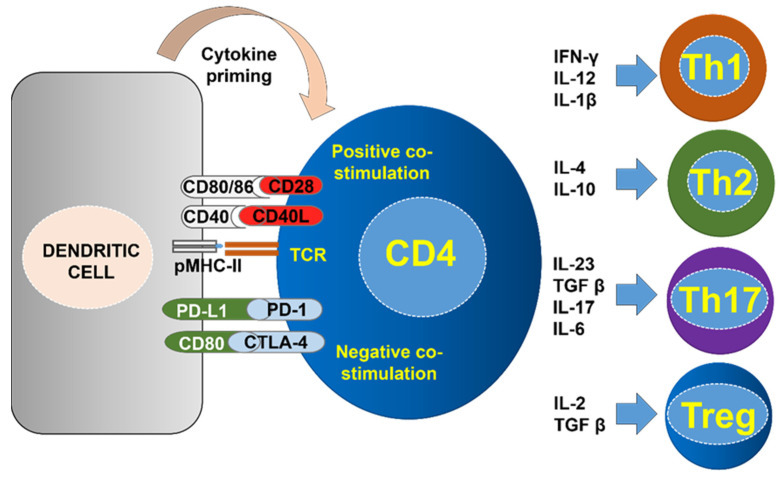
Three signal model of antigen presentation to T cells and differentiation into CD4 T helper subtypes. On the left, a dendritic cell presents antigenic peptide complexes with MHC-II to the CD4 T cell. TCR recognition drives signal 1. Then, CD4 T cells receive both positive co-stimulation and negative co-stimulation as shown in the figure. Some representative receptor-ligand interactions from both types of co-stimulation are shown in the figure. The integration of these signals results in distinct activation degrees. A third cytokine signal is also provided by the dendritic cell which determines the differentiation fate of the CD4 T cells. On the right, examples of cytokines which drive CD4 T cell differentiation to the main T helper subtypes as shown. Regulatory T cells can also be differentiated from uncommitted CD4 T cells as shown in the figure.

CD4 T cells differentiate into several subsets with different regulatory roles. This diversity of CD4 T cell subsets reflects the variety of immune responses they regulate [70]. CD4 T cells differentiate during antigen presentation towards different subtypes depending on the cytokine milieu (Figure 2). During antigen presentation, mostly at peripheral lymph nodes, antigen-presenting cells such as DCs present antigen peptides complexed to MHC-II molecules. These MHC-II-peptide complexes are recognised by naïve CD4 T cells. In this process, CD4 T cells receive three signals; the first through their TCR, the second the integration of positive and negative co-stimulation. The third one is called cytokine priming, and consists on stimulation by cytokines produced by antigen presenting cells and those present in the microenvironment. The specific cytokines determine the CD4 T helper (Th) subtype that will be activated. The major Th subtypes are Th1, Th2, Th17 and inducible regulatory T cells (Tregs) (Figure 2).

**Figure 3:**
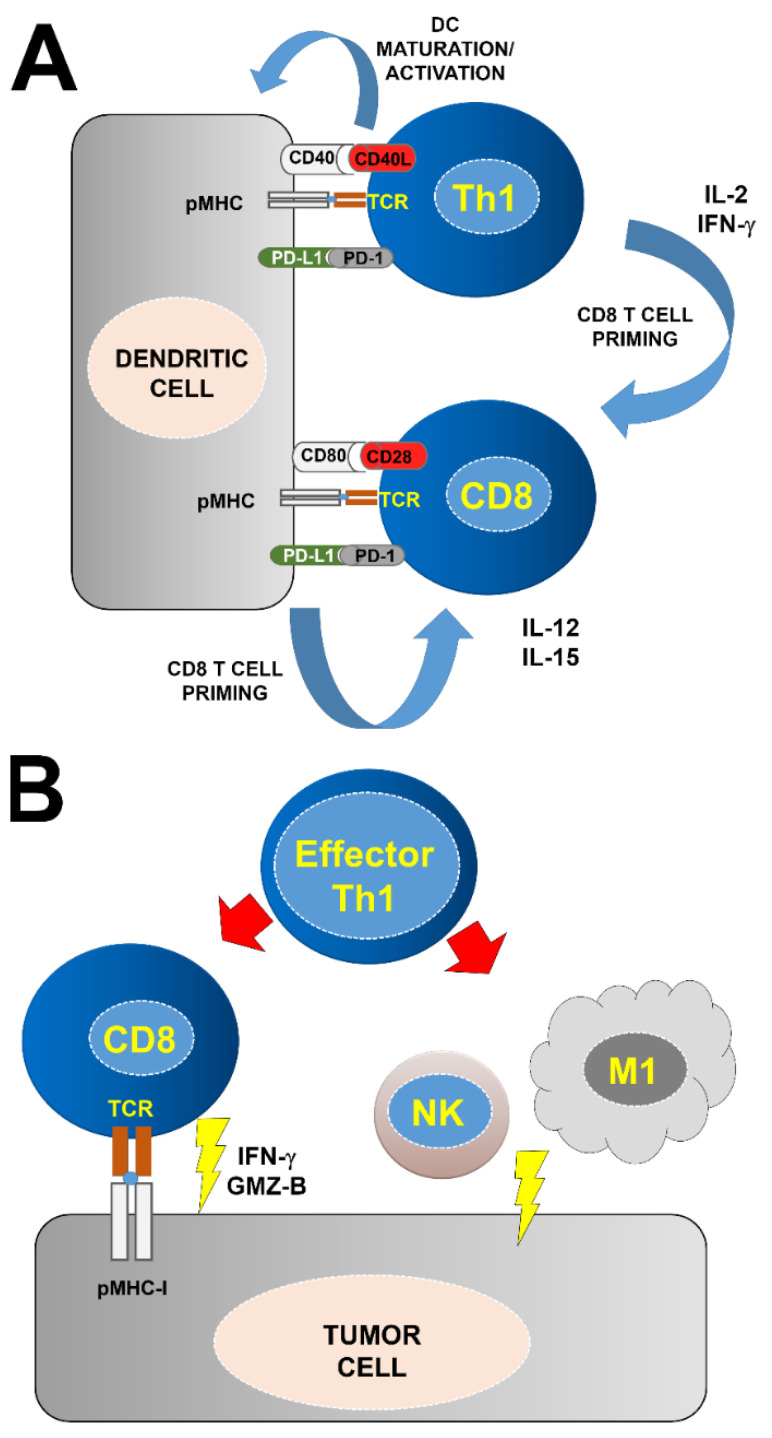
Role of CD4 Th1 cells in anti-tumor immunity. The best characterized mechanisms by which CD4 Th1 cells exert in anti-tumor responses are schematically shown in the figure. (**A**) Dendritic cells present antigenic peptides complexed to MHC molecules (pMHC) on their surface to both CD4 Th1 cells (top) and naïve CD8 T cells (bottom). CD4 Th1 cells participate in the appropriate priming of CD8 T cells by enhancing dendritic cell maturation and activating CD8 T cells through cytokine priming. The main co-stimulatory interactions are shown for CD4 and CD8 T cells. PD-L1 binding to PD-1 on the T cell surface is shown. This interaction also takes place during physiological antigen presentation, and it regulates ligand-induced TCR down-modulation. These processes are termed DC and CD8 licensing. (**B**) In a second step, CD8 CTLs infiltrate tumors and attack tumor cells after TAA recognition through a variety of mechanisms. The production of IFNs and granzyme are shown just for illustration. Tumor-infiltrating Th1 cells can directly activate NK and M1-macrophages enhancing their innate anti-tumor responses. NK cells can directly lyse tumor cells, and antigens can be picked up by M1 macrophages, which further enhance antigen presentation and activation of CD8 T cells. Th1, T helper 1; NK, natural killer; M1, type-1 macrophages.

Classically, the CD4 Th1 subtype is associated to anti-tumor immunity (Figure 3). Th1 cells act in concert with antigen presenting cells for CD8 T cell priming and differentiation towards CTLs in a process called T cell licensing (Figure 2) [71,72]. During this process, Th1 cells and APCs produce pro-inflammatory cytokines such as IFNγ and IL-12 [73,74,75]. Th1 cells are also responsible for DC licensing through engagement of CD40L with CD40 on the DC surface [76,77,78]. During this process, DCs mature by up-regulating the co-stimulatory molecules CD80, CD86 and CD40L, together with concomitant IL-12 and IL-15 secretion for cytokine priming [79,80,81,82,83,84]. Activated DCs prime naïve CD8 T cells towards CTLs and memory phenotypes (Figure 3). CD8 T cells acquire CTL effector functions through co-stimulation by CD27 with CD70 via CD40-CD40L signaling between APCs and CD8 T cells [65,85,86]. Indeed, CD8 T cell priming in the absence of CD4 T fails to fully activate CTLs leading to limited expansion of anergic CD8 T cells with dysfunctional phenotypes, and lack of CD8 T cell memory [65,72,87,88,89]. The activity of CD4 Th1 cells potentiates anti-tumor responses from NK and M1-type macrophages, which further promotes tumor killing causing the release of more TAAs for T cell priming [90,91]. CD4 T helper cells can also differentiate into Th2 and Th17 subtypes, characterized by expression of cytokines such as IL10 and IL4 for the former, and IL-6, IFN-γ and IL-17 for the latter (Figure 2) [92,93,94,95,96]. These T helper subtypes are generally associated with tumor progression, although this may be context-specific. For example, CD4 Th2 cells are required for long-term memory responses, while Th17 cells can induce potent inflammation that can amplify anti-tumor immunity [97,98,99,100,101,102]. Regulatory CD4 Tregs consist of at least three main subtypes, natural Tregs, inducible Tregs, and Tr1 CD4 T cells that are involved in maintaining central and peripheral tolerance. These CD4 T cells present potent immunosuppressive activities by several mechanisms, ranging from cell-to-cell contacts and expression of immunosuppressive cytokines [103,104,105,106,107,108,109,110]. In certain conditions, CD4 T cells can also acquire direct cytotoxicity through production of IFN-γ and TNF-α, expression of FasL and TRIAL, and cytotoxic granules [111,112,113].

## 3. CD4 T Cell Differentiation Phenotypes according to Effector Functions

Upon antigen presentation, both CD8 and CD4 T cells expand exponentially and differentiate into effector phenotypes, including CTLs and the different subsets of T helper cells. These T cells are short-lived, but a small pool survives as long-lived memory subsets after antigen clearance. These memory T cells can last decades and are critical for recall responses. Memory T cells need less requirements for activation and mobilization following antigen presentation compared to naïve T cells. Following antigen re-encounter, memory T cells undergo fast activation and expansion, leading to stronger effector T cells [114,115,116,117]. Different T cell differentiation phenotypes can be readily distinguished in humans by evaluating the expression profiles of CD62L and CD45RA. CD62L+ CD45RA+ naïve T cells migrate out of the thymus towards secondary lymphoid organs by the expression of CD62L [117]. Memory T cells can be differentiated into two main types based on their location and migration patterns: Memory T cells residing in secondary lymphoid organs are represented by central memory subsets, while those migrating into sites of inflammation are termed effector memory T cells. As such, memory T cells lose CD45RA expression, which allows these subsets to move between secondary lymphoid organs. Effector memory T cells further lose CD62L expression, as these cells will remain tissue-resident and upregulate chemokine and cytokine receptors required for chemotaxis towards sites of inflammation. From the effector memory pool, T cells can then re-express CD45RA (effector memory cells that re-express RA, or EMRA), which is also a marker of terminal differentiation. EMRA cells end up accumulating during the lifetime of the individual [118].

In addition to this classification, human T cell differentiation can also be studied based on CD27/CD28 expression profiles. Thus, poorly differentiated T cells which includes naïve and central memory phenotypes co-express both markers. Then, T cells progressively lose first CD27 expression, and then CD28, leading to highly differentiated T cells which englobe effector memory and EMRA T cells [29,119,120,121,122]. CD27- and CD28- CD4 T cells are considered senescent T cells in humans.

## 4. CD4 Immunity in PD-L1/PD-1 Blockade Immunotherapy

PD-L1/PD-1 blockade immunotherapies consist of systemic administration of recombinant monoclonal antibodies that disrupt PD-L1/PD-1 interactions. These antibodies do not specifically target this interaction at the tumor site. Therefore, the specific cellular and molecular mechanisms behind clinical responses are under investigation. PD-L1 is frequently overexpressed by cancer cells and tumor-associated cells. These include immune cells of the myeloid lineage, such as macrophages, dendritic cells and myeloid-derived suppressor (MDSC) cells [7,19,123,124]. In cancer cells, PD-L1 also confers resistance to cancer cells from pro-apoptotic signals and enhances their proliferation [18,28,125,126,127]. Tumor-infiltrating T cells are frequently inactivated within the tumor microenvironment through PD-L1/PD-1 interactions [128]. The main effector mechanism of PD-L1/PD-1 blockade seems to be the reversal of T cell dysfunctionality by interfering with PD-1 signals within the T cells, and reactivating key TCR-dependent signaling pathways [18,22,28,126,129,130,131,132] (Figure 1). For example, tumor-infiltrating PD-1^high^ CD8 T cells proliferate in patients under PD-L1/PD-1 blockade [133,134,135]. Indeed, we previously showed that apart from recovering TCR signaling, interference with PD-1 signaling in T cells prevents CBL-b up-regulation [7], an E3 ubiquitin ligase of the casitas B cell lymphoma (CBL) family (Figure 1). These E3 ubiquitin ligases are major regulators of TCR expression, and TCR intracellular signaling [136,137,138,139,140]. Inhibition of CBL-b expression caused a proliferative burst in CD8 T cells [7,16,83]. Nevertheless, tumor-infiltrating PD-1+ CD8 T cells present different degrees of exhaustion, and are phenotypically highly heterogeneous [46,55,141,142,143]. This heterogeneity also affects their susceptibility to be reactivated by single blockade of the PD-L1/PD-1 pathway.

Increasing experimental evidence indicates that the PD-L1/PD-1 blockade within the tumor microenvironment is not sufficient. Systemic immunity may play a relevant contribution to efficacy. Several groups have reported that there is a systemic proliferative burst of PD-1+ T cells following immunotherapies [29,46,57,134,135,144,145,146]. Recently, it has been shown that most of the expanded effector CD8 T cells after PD-L1/PD-1 blockade are novel clonotypes which arose in the periphery rather than re-activated from within the tumor [47]. In non-small cell lung cancer (NSCLC) patients undergoing PD-1 blockade monotherapies, systemic expansion of PD-1+ CD8 T cells correlated with efficacy [57,144,145]. These T cells corresponded to effector phenotypes but with up-regulated CD27, CD28 and ICOS, markers related to poorly differentiated T cells. However, the authors of these studies did not show the specificity of these expanded clonotypes, although they were very likely TAA-specific. Similar results were obtained in melanoma patients treated with anti-PD-1 immunotherapy [146]. In this case, the expanded PD-1+ CD8 T cell clones corresponded to those infiltrating the tumor. Similar results were reproduced in which changes in systemic memory CD8 T cells correlated with efficacy [147].

If we consider that systemic expansion of effector CD8 T cell clones may play a key role in the efficacy of PD-L1/PD-1 blockade immunotherapies, it is highly likely that CD4 T cells regulate this expansion through helper functions (Figure 3). Indeed, there is increasing experimental evidence demonstrating the importance of CD4 immunity for cancer immunotherapy [148,149,150]. An immunotherapy treatment based on administration of anti-cancer cell immunoglobulins showed efficacy following the expansion of a CD4 T cell subset characterized by expression of CD44, CXCR3, ICOS and the transcription factor T-bet [148,151,152]. Similarly, melanoma responder patients undergoing anti-PD-1 therapy showed a systemic increase in a CD4 T cell subset compared to non-responders [148]. The importance of CD4 responses in anti-tumor immunity was highlighted by the need for CD4 tumor-derived neoepitopes for efficacious immunotherapy [68,153,154,155]. In mouse models of cancer, MHC-II-restricted epitopes derived from these neoantigens were also required for anti-tumor immune responses [156].

Our research group and others have characterized the contribution of systemic CD4 immunity to the efficacy of PD-L1/PD-1 blockade immunotherapy. The combined data independently demonstrate that the status of systemic CD4 immunity before starting immunotherapies is a critical factor for clinical responses in NSCLC. Indeed, we showed that elevated CD4 memory T cells with highly differentiated phenotypes developed objective responses to PD-L1/PD-1 blockade [29,122]. These T cells in responder patients retained relatively high proliferative capacities, which were further augmented following PD-1 blockade ex vivo and in vivo. Moreover, these T cell subsets showed reduced co-expression of PD-1 and LAG-3 immune checkpoint molecules. On the other hand, patients with reduced numbers of memory CD4 T cells in peripheral blood failed to respond to PD-L1/PD-1 blockade. Their CD4 T cells showed strong dysfunctionality and failed to proliferate. This dysfunctionality was accompanied by strong up-regulation of both PD-1 and LAG-3 simultaneously within the same cells, leading to an anergic state. Ex vivo, PD-1 blockade failed to stimulate their proliferation, most likely due to the concomitant expression of LAG-3 [44]. As CD8 T cells are key effector anti-tumor molecules, we evaluated the functional status of systemic CD8 T cells. Both responders and non-responders exhibited highly dysfunctional CD8 T cells before starting immunotherapies. However, only in patients with functional CD4 T cells, PD-L1/PD-1 blockade achieved reactivation of systemic CD8 immunity. Highly dysfunctional CD4 T cells from non-responder patients recovered their proliferative activities ex vivo by PD-1 and LAG-3 co-blockade [21]. It has to be mentioned that PD-1/LAG-3 co-blockade is demonstrating significant clinical improvement for the treatment of melanoma, having been approved recently by the FDA [43]. Nearly at the same time, the team led by Kobayashi identified a similar CD62^low^ effector memory CD4 T cell subset in responder NSCLC patients which was enriched in patients before starting immunotherapy [50]. These patients were long-term responders, and those who acquired resistance to treatment showed a decrease in this T cell population. A transcriptomic profile uncovered that these T cells corresponded to TCR-activated Th1 cells with potential to activate CTL responses. This latter study indicated that these systemic CD4 T cell responses had to be maintained over time for long-term responses. The study by Kagamu and collaborators, together with our study, provides strong evidence that functional CD4 immunity is needed to achieve efficacious clinical responses in NSCLC [48]. While this manuscript was in revision, another study corroborated the utility of systemic CD4 T cell quantification as a biomarker of response to immunotherapy in patients from the landmark study KEYNOTE-001 [157].

Overall, all the combined evidence reflects the need for functional CD4 memory responses in PD-L1/PD-1 blockade immunotherapies, but the cellular and molecular mechanisms underlying this requirement are still poorly understood. To acquire full cytotoxic activities, CD8 T cells need licensing from both antigen presenting cells and CD4 T cells through cell-to-cell interactions and cytokine priming (Figure 2 and Figure 3) [8,71,78,81,82,83,84,158,159,160,161,162,163]. As such, APCs simultaneously present antigens to both CD8 and CD4 T cells, and CD4 T cells in turn produce cytokines that stimulate further APC maturation and CD8 T cell activation. In a recent study, we evaluated the status of systemic myeloid immunity, as myeloid cells regulate CD4 and CD8 activation through antigen presentation among other mechanisms. Interestingly, NSCLC patients with high diversity of myeloid cell types in peripheral blood before starting PD-L1/PD-1 blockade immunotherapies responded to the treatment [45]. These patients exhibited an enrichment in HLA-DR+ monocytic subsets, and a significant decrease of granulocytic cells, including neutrophils and granulocytic-MDSCs. Therefore, these data show that, most likely, a functional myeloid compartment could be important to maintain functional CD4 immunity, and this in turn will activate CD8 responses towards cancer cells [45].

Another different issue is whether tumor infiltration with CD4 T cells plays a role in anti-tumor immunity. A recent study favors this model as well, both in mouse models and in tumor biopsies from classic Hodgkin lymphoma for PD-1 blockade [164]. It may be possible that CD4 infiltration may favor subsequent CTL infiltration in PD-L1/PD-1 blockade immunotherapies [165]. On the other hand, accumulation of PD-1^high^ CD4 subsets within the tumor correlated with tumor burden, as shown in a clinical study with NSCLC [166]. Reduction of Tregs in these patients was a correlate of increased overall survival. Considering all the published data, it seems that exhausted T cells infiltrating the tumor are probably not the main target of PD-L1/PD-1 blockade, but rather systemic T cell clones which get activated and infiltrate the tumor following therapy.

## 5. Systemic CD4 Immunity as a Biomarker of Response to PD-L1/PD-1 Blockade Immunotherapy

Biomarkers of response to immunotherapies have typically been looked for within the tumor, or by evaluating tumor infiltration by immune cells. This is also the case for PD-L1/PD-1 blockade. For the latter case, the assessment of PD-L1 expression in tumor biopsies is the most well-established. Nevertheless, other markers including neoantigen expression, mutational status, DNA repair proteins and infiltration with TILs and immunosuppressive cells have been used. PD-L1 expression in tumors is the most extensively used, but its reliability could be a function of the tumor type, and also of the specific PD-L1/PD-1 blockade drug used [18,125,167,168,169]. Tumor mutational burden and transcriptomic analyses of TILs can also have predictive value in patients undergoing PD-L1/PD-1 blockade [170,171,172,173]. It is evident that a major drawback in implementing these biomarkers in clinical practice is, apart from their price, the unavailability of sufficient material from tumor biopsies in many cases. A second issue is whether a sample represents the tumor heterogeneity found in patients. Indeed, PD-L1/PD-1 blockade is carried out systemically and will have a broad impact in the immune system. These global effects will probably contribute to clinical responses, and systemic immune biomarkers could be an inexpensive way forward for their clinical use [19,29,45,48,50,57,144,174,175]. Therefore, immune profiling of immune cells in peripheral blood samples could be a promising non-invasive procedure to evaluate biomarkers of response in immunotherapies. Profiling studies in combination with tumor sampling could improve patient stratification for immunotherapies. For example, early expansion of PD-1+ CD8 T cells in peripheral blood by flow cytometry correlated with clinical efficacy, which was confirmed in patients with thymic epithelial tumors [57,144]. In the first study, T cell expansion was evaluated by assessing proliferation through the expression of Ki67 after the first week of treatment. The predictive value of this functional immunological biomarker was validated in two independent cohorts of NSCLC patients. Similarly, genome-wide sequencing of peripheral T cell populations uncovered early clonal expansion that was associated with clinical responses in NSCLC patients undergoing PD-1 blockade [176]. This phenomenon seems to be common to many tumor types, for example in metastatic melanoma patients treated with PD-1 blockers [146]. High-dimensional techniques for the analysis of multiple populations can increase the accuracy for the identification of cellular biomarkers of response. For example, the identification of a CCR7-CD27-CD8 T cell subset that expanded systemically after PD-1 blockade in melanoma patients [147]. All these data clearly indicate that quantification of proliferating CD8 T cell populations following PD-L1/PD-1 blockade may be suitable biomarkers of response, or at least as a biomarker for real-time monitoring of responses in patients using a non-invasive technique. These biomarkers have the advantage of helping the clinician in decision-making during the early onset of immunotherapies. However, they may not be useful for the early identification of hyperprogressors [31]. Therefore, quantification of CD8 T cell subsets before the start of immunotherapies may not be sufficient for the identification of responders and non-responders. Other high-dimensional techniques with the power of identifying multiple immune cell populations have been applied for the identification of predictive biomarkers. For example, mass cytometry (CYTOF) which has been applied for the analysis of peripheral blood populations in metastatic melanoma patients undergoing PD-1 blockade before treatment initiation. One pioneering study showed that elevated CD14+ CD16- HLA-DR^high^ monocytes before applying immunotherapy correlated with a significant increase in progression free survival (PFS) [177]. In agreement with this study, we found a highly significant association between the baseline percentage of HLA-DR+ CD14+ monocytic cells and objective responses in NSCLC patients under PD-L1/PD-1 blockade by high-dimensional flow cytometry in peripheral blood [45]. Our results confirmed the predictive power of this immune cellular biomarker. Another study demonstrated that elevated numbers of CD69+ MIP-1b+ NK cells were significantly associated with responders [178]. Again, we showed that NK cells were, indeed, key effector cytotoxic cells that were associated to high myeloid diversity and increased monocytic cells [45]. A recent study corroborated the predictive power of CD4 T cells, NK cells and monocytes in a cohort of patients from the seminal KEYNOTE-001 study [157]. The application of multi-parametric flow cytometry highlighted the elevation of PD-1, PD-L1 and PD-L1 in T cells as biomarkers of worse survival [179]. However, this might not apply to myeloid cell populations, in which elevation of PD-L1 correlate with efficacy of PD-L1 blockade with atezolizumab in NSCLC patients with PD-L1-negative tumors [19]. Nevertheless, none of the above immune cell-based biomarkers have been fully validated in prospective studies. In addition, some of the high-dimensional technologies such as CYTOF are difficult to standardize and apply into routine clinical practice.

Work from independent research groups is demonstrating the potential of quantifying CD4 T cell subsets in peripheral blood to predict the efficacy of PD-L1/PD-1 blockade immunotherapies. In a small-scale study in metastatic melanoma patients, elevation of central memory T cells was associated with prolonged survival [180]. A higher ratio of central memory versus effector T cell subsets correlated with benefit from PD-1 blockade in a small cohort of NSCLC patients [181]. Two prospective studies, including our own, independently showed that evaluating the dynamics of CD4 T cell populations in peripheral blood can predict clinical outcome in patients treated with PD-L1/PD-1 blockade immunotherapy [29,48,50,175]. Both studies independently found similar quantitative thresholds for evaluation of CD4 memory T cells in peripheral blood before the start of immunotherapies. In our study, CD4 T cells exhibited a CD27-CD28^low^ phenotype, which included both enriched central and effector memory CD4 T cell subsets [29]. Response rates of about 50% were observed in patients with more than 40% of the T cells with this biomarker phenotype. All patients with percentages below this threshold were non-responders and with a significant increase in risk for developing hyperprogression [29,30]. Kagamu and collaborators independently found a population of CD4 T cells (CD62L^low^ effector memory subset) with similar threshold values as described by us [48,50]. Elevation of this subset and reduction in Tregs were significantly represented in responders to PD-1 blockade immunotherapy. A high ratio between effector memory CD4 T cells and Treg cells could identify responders from progressors.

Other immunological biomarkers have also been studied in many cancer types and for a variety of treatments, including immunotherapies and PD-L1/PD-1 blockade. These include absolute and relative neutrophil and lymphocyte numbers, and the classical neutrophil-to-lymphocyte ratio (NLR), amongst others [182,183,184,185]. These markers have been shown to have prognostic rather than predictive power. This is a subtle difference, but whereas a prognostic marker is treatment-independent, a truly predictive biomarker is one specifically associated with a particular therapy. However, the quantification of these biomarkers is not standardized, and their practical value might be limited [45]. Modifications to the classical NLR have been introduced to incorporate other immune populations, especially other myeloid cells apart from neutrophils. For example, the derived NLR (dNLR) that together with quantification of lactate dehydrogenase is used for the calculation of the lung immune prognostic index for PD-L1/PD-1 blockade [186]. However, all these classical prognostic markers are based on standard clinical blood analyses that do not discriminate the variety of immune cell types and their activation status in peripheral blood [45]. To achieve this, high-dimensional techniques, such as multiparametric flow cytometry, can provide more accurate quantification of different cell types, leading to better correlations with clinical outcomes. Using these techniques, the prognostic value of cell populations such as monocytes, neutrophils and other granulocytes have been confirmed in NSCLC patients undergoing PD-L1/PD-1 blockade immunotherapies [45,174].

## 6. What the Future Holds

Most investigations have been centered in the tumor microenvironment. This is an obvious choice because all the effector mechanisms and tumor killing takes place within the tumor. This is not an exception with PD-L1/PD-1 blockade immunotherapies. However, these therapies imply the systemic and periodic administration of anti-PD-L1 and anti-PD-1 antibodies at high concentrations. Hence, systemic and pleiotropic effects should be expected that could influence clinical benefit. As we have discussed in this review, in recent years more studies have evaluated the impact of these immunotherapies over systemic immunity, and the changes in immune cell populations within the cancer patient that may have a significant contribution to clinical efficacy. It is true that many of these studies are difficult to validate independently. Part of this is caused by the complexity of some analytical techniques and the associated high costs. Some of the classical prognostic markers based on quantification of immune cell populations from standard blood analyses also lack predictive capacities, and are highly dependent on the manipulations implemented by the specific hospital. Moreover, these analyses lack the power to discriminate the complexity and diversity of immune cell types in peripheral blood. This is the case with myeloid cell types. On the other hand, T cells may show less variability in subtypes and could be more amenable for quantification. Several of these studies have monitored the dynamics of CD8 T cell populations during PD-L1/PD-1 blockade immunotherapies, demonstrating value in identifying responders [57,145,146]. As CD8 T cell activation and expansion depends on antigen presentation and licensing by CD4 T cells and myeloid cells, emerging studies are indicating that a functional systemic CD4 and myeloid immunity is a pre-requisite for clinical responses to immunotherapies [29,45,50]. Peripheral CD4 memory T cells seem to be a reliable biomarker for immune profiling. This is supported by independent studies with nearly identical results. Even if they are not used as practical predictive biomarkers, the “real-time” quantification of T cell dynamics from peripheral blood samples could help clinicians to identify patient populations that are responding, or those at risk of developing hyperprogressive disease [30,48,49].

PD-L1/PD-1 blockade immunotherapies seem to reinvigorate systemic immunity, particularly the activation and expansion of CTLs, which might be orchestrated in the periphery by myeloid antigen-presenting cells and CD4 T cells. This fact suggests that overcoming the strong T cell dysfunctionality observed in many cancer patients could be a way forward before administering PD-L1/PD-1 blockade. It has to be remarked that part of the strong dysfunctionality observed in both CD4 and CD8 T cells is caused by co-expression of multiple immune checkpoint molecules. An example is PD-1 with LAG-3 [21,29,187]. LAG-3 expression in CD4 and CD8 T cells together with PD-1 conferred resistance to PD-L1/PD-1 blockade. PD-1/LAG-3 co-blockade ex vivo and in mouse models reversed CD4 dysfunctionality [21,29,187,188]. In March 2022, Opdualag was approved as a first line treatment for melanoma. This treatment consists of a combination of anti-PD-1 nivolumab and anti-LAG-3 relatlimab [43,189,190,191]. This is the first time that a LAG-3 blocker has been approved for clinical use. This treatment presents acceptable toxicity and an evident improvement over monotherapies.

The remarkable results from immune checkpoint inhibitor immunotherapies have re-opened the gate for extensive research in cancer immunotherapy. While development of immune checkpoint blockers and adjuvant therapies have rocketed up, there is an increasing need in understanding the immunological bases of clinical responses, and identification of adequate biomarkers allowing correct patient discrimination.

## Figures and Tables

**Table 1:** FDA-approved PD-L1/PD-1 blocking antibodies.

Inhibitor	Target	Approval	Cancer Types
Nivolumab	PD-1	2014	Non-small cell lung cancer, melanoma, head and neck cell cancer, urothelial carcinoma, renal cell carcinoma, classical Hodgkin lymphoma, microsatellite, instability-high solid cancer, gastric cancer, cervical cancer, hepatocellular carcinoma, Merkel cell carcinoma, primary mediastinal large B-cell lymphoma
Pembrolizumab	PD-1	2014	Small cell lung cancer, non-small cell lung cancer, melanoma, urothelial carcinoma, colorectal cancer, hepatocellular carcinoma, advanced renal cell carcinoma, classical Hodgkin lymphoma, head and neck squamous cell cancer.
Atezolizumab	PD-L1	2016	Urothelial carcinoma, non-small cell lung cancer, small cell lung cancer, triple-negative breast cancer.
Avelumab	PD-L1	2017	Locally advanced or metastatic urothelial carcinoma, metastatic Merkel cell carcinoma.
Durvalumab	PD-L1	2017	Locally advanced non-small cell lung cancer, small cell lung cancer, metastatic urothelial carcinoma.
Cemiplimab	PD-1	2018	Locally advanced and metastatic cutaneous squamous cell carcinoma.
Dostarlimab	PD-1	2021	Endometrial cancer.
